# Magnitude and Associated Factors of Psychoactive Substance Use among Youths at Selected Administrative Towns of North Shewa Zone, Amhara Region, Ethiopia

**DOI:** 10.1155/2023/2124999

**Published:** 2023-04-03

**Authors:** Elyas Admasu Basha, Aklil Semu Tefera, Alemnesh Tesema Tilahun, Asrat Fenta Amede

**Affiliations:** ^1^Department of Nursing, College of Health Science, Debre Berhan University, Debre Berhan, P.O. Box 445, Ethiopia; ^2^Department of Medicine, College of Medicine, Debre Berhan University, Debre Berhan, Ethiopia; ^3^Department of Laboratory, College of Medicine, Debre Berhan University, Debre Berhan, Ethiopia; ^4^Department of HIT, Debre Berhan Health Science College, Debre Berhan, P.O. Box 37, Ethiopia

## Abstract

Youth and adolescent psychoactive substance use is a major public health problem worldwide which affects the health of individuals, families, and the community as a whole. This study was aimed at assessing the level of psychoactive substance use and the associated factors among the youths of North Sowa zone, Amhara region, Ethiopia, 2020/21. Community based cross-sectional study design with a multistage sampling technique was used. A total of 632 youths were involved in the study. Linear logistic regression analysis was used for both bivariate and multivariate analysis. The prevalence of life time and current psychoactive substance use was found to be 66.1% and 65%, respectively. Male sex and history of substance use from family members and friends were significantly associated with psychoactive substance use. In this study, the prevalence of life time and current psychoactive substance use was high as compared to other studies conducted in Ethiopia.

## 1. Introduction

Substance/drug is a licit or an illicit chemical agent if taken into the body is capable of causing physiological and psychological changes [1]. Tobacco, alcohol, and illicit drugs are among the top 20 risk factors for ill health identified by the World Health Organization (WHO) [2]. Psychoactive substances can be classified into CNS stimulants, depressants, and hallucinogens [3]. There are many types of disorders occurred due to the use of these substances. According to the DSM-V, substance related disorders are categorized as substance use disorders, substance induced intoxication and withdrawals, and substance induced mental disorders. Substance use disorder is a maladaptive pattern of substance use leading to a significant impairment or distress [4]. All age groups, both sexes, and all people in any socio-economic status can be affected by substance use [5]. However, youths (adolescents) are more vulnerable for substance use problems [6–8]. Human brain development started within the uterus immediately after fusion of egg and sperm and continues until the early adulthood. This range of time is critical to human mental health that any offending things including psychoactive substance can affect normal development. The cognitive part of the brain which is mainly responsible for controlling impulses, thinking, planning and decision making and are immature enough when the individual reaches at least the age of 18 years to recognize the useful and harmful behaviors. This immaturity predisposes them to experiment new behaviors whether it is harmful or useful due to lack of judgment. The same is true for psychoactive use [9, 10].

Psychoactive substance use of adolescents causes an enormous burden on individuals, families, and communities. Youth and adolescent psychoactive substance use is a major public health problem facing the world today [11]. Most adolescents start using drugs as young as 12 years of age [12]. Peer pressure, family problems, availability of substances, and getting relief from their stress are some of the contributing factors to start their substance use [13]. These adolescents seem not to be considering the long-term effect of these drugs on their lives. The use of drugs by youths and adolescents also affects their academic performance. Some of these adolescents end up dropping out of school and adding to the rising rate of unemployment [14, 15]. Substance use among adolescents and youths ranges from experimentation to severe substance use disorders. In addition to long-term effects of psychoactive substance use, there might be immediate problems faced by youths, such as accidents, fights, unwanted sexual activity, and overdose [16–18].

Substance use also interferes with adolescent brain development. Adolescents are vulnerable to the effects of substance use and are at increased risk of developing long-term consequences, such as mental health disorders, underachievement in school, a substance use disorder, and higher rates of addiction, if they regularly use substances/drugs during adolescence and youth [19].

The use of psychoactive substance is prevalent throughout the world and is associated with a significant public health burden [20]. It is estimated that tobacco is responsible for 8.8% of all deaths and for 4.1% of the global burden of all diseases, which is measured as the number of years spent living with a disease, while alcohol is responsible for 3.2% of deaths and 4% of disability-adjusted life years (DALYs). Excessive alcohol use and other substance abuse are also risk factors for a wide variety of social, financial, legal, and relationship problems for them and their families. Globally, there is an increasing trend for people to use multiple substances [2, 21].

Even though the public impact of substance use among youths is paramount globally, there is little information found from community based studies about the prevalence and associated factors of substance use in this age group especially in our country, Ethiopia. By searching for the exact community based magnitude of psychoactive substance use and its associated factors among youths will have an entry point to future intervention program on it. Therefore, our current community based study was aimed to assess the prevalence and factors associated with psychoactive substance use among youths aged 15–24 years old.

### 1.1. Hypotheses Development

The bio-psychosocial model provides the explanation about the cause of substance use [22]. According to this model, biological, psychological, and social factors interact to cause psychoactive substance use [22, 23]. Being children of alcohol-dependence parents can most probably use psychoactive substance. Individuals who are genetically predisposed to substance use disorder enter the world with a greater risk of becoming addicted at some point in their lives [24]. Adolescents constitute the most important risk group in substance use [25]. According to a report published by UNODC (United Nations Office on Drugs and Crime), age interval of first substance use is approximately 11 up to 17 years old worldwide [26].

Psychosocial components are other contributing factors to the risk of addiction along with biological (genetics). Disruption of normal child-parent relationships, lack of involvement in organized groups, and few effective peer relationships may have been predisposing factors in some individuals initiating use of psychoactive substance. An individual exposed to drug use at an early age can also be influenced by social modeling (or learning via observation). Socialization to nontraditional norms, parental modeling of licit and illicit drugs uses, and involvement with drug-using peers may have been important factors in initial use for other individuals [27]. We concluded that the following hypotheses are proposed to be proved by our study.

  Hypothesis 1 (H1): Young people are more vulnerable to psychoactive substance use than older adults.
  Hypothesis 2 (H2): Being children of psychoactive substance-dependence parents are more likely to use psychoactive substance.
  Hypothesis 3 (H3): There is no difference between psychoactive substance use and participants' age, parent modeling, and peer pressure.

## 2. Methods

### 2.1. Study Area and Period

This study was conducted from January 1, 2020 to March 30, 2020 at selected administrative towns, North Shewa zone, Amhara region, Ethiopia. North Shewa zone has 5 administrative towns. Based on the 2007 Census conducted by the Central Statistical Agency of Ethiopia (CSA), this Zone has a total population of 1,837,490. The five administrative towns have 62,461 young people.

### 2.2. Study Design

Community based cross-sectional design with quantitative methods was used.

### 2.3. Study Population

All youths age 15–24 years old at selected administrative towns, in North Shewa zone, Ethiopia, were involved in the study.

### 2.4. Operational Definitions

#### 2.4.1. Psychoactive Substance

A substance or drug when taken to the body that has the ability to change the function of CNS.

#### 2.4.2. Life Time Substance Use

When an individual takes any one of the psychoactive substances at least once in his/her life.

#### 2.4.3. Current Substance Use

When an individual takes any one of the psychoactive substances at least once in the last 3 months.

### 2.5. Sample Size and Sampling Technique

The sample size was determined by an assumption of 70% which was from Adama town, 5% marginal error and 95% confidence interval of certainty (alpha = 0.05). Based on this assumption, the actual sample size for the study was computed using single population proportion which resulted in 323. Since we used multistage sampling technique to find our samples, we multiplied the calculated sample size by a design effect of 2. So, our final sample size was 646. Among the five administrative towns three were selected by lottery method. The participants were allocated proportionally for each study area i.e., 240 for Debre Berhan town, 206 for Alem ketema, and 200 for Shewarobit town. The second stage was kebeles from each administrative town at which 4 kebeles from Debre Berhan, 3 kebeles for each of Alem ketema and Shewarobit administrative towns selected randomly by lottery methods. Finally, the selected kebeles were clustered and the data were collected as a house hold level.

### 2.6. Data Collection Instruments

English version of the interviewer administered ASSIST (alcohol, smoking, and substance involvement screening test) questionnaire validated by the WHO was used. Structured questionnaires for other factors reviewed from different literatures were prepared. The ASSIST has 8 items which is an easy-to-use tool that detects substance use and related problems. Since the questionnaire was developed by the WHO, it is considered as reliable and valid to be used by any country [28]. It was translated to Amharic to collect the data and again it was translated back to English to check for its consistency and analysis by experts. The questionnaire was pretested in one of the town districts which were not included in real data collection. The pretest was conducted on 5% of the total sample size. Then, the questionnaire was reassessed for its clarity, length, and completeness. The Cronbach's alpha coefficient of ASSIST was 79.4%.

### 2.7. Data Analysis

The questionnaires were checked by the principal investigator for completeness. Unfilled and partially filled questionnaires were excluded. The remaining were coded, cleaned, and entered into EPI data version 3.1. Then, the data were transferred into and analyzed by statistical package for social sciences software package (SPSS) version 21. In our data analysis procedure the chi-square and *G* *∗* power analyses were conducted.

The descriptive analysis such as frequency distribution and percentages were calculated. Odds ratio with 95% confidence interval was used to ascertain the association between dependent and independent variables. Bivariate and multivariate analyses were used to identify independent predictors of current substance use among the youths. Confidence interval of 95% was also used to see the precision of the study and level of significance.

### 2.8. Ethical Considerations

Ethical clearance was obtained from IRB (institutional review board) of Debre Berhan University (DBU), institute of health science and medicine. Then, formal letter of cooperation was written to North Shewa youth and sport affair Bureau. Oral informed consent was obtained from each participant after each study participant was adequately informed about the purpose, method, anticipated benefit of the study, and their full right to discontinue or refuse to participate in the study by the data collectors. No need of asking/writing the names of the participants to keep confidentiality and cultural norms were respected properly.

## 3. Results

### 3.1. Socio-Demographic Characteristics

Out of the expected 646 respondents, 632 questionnaires were correctly and completely filled in the study, yielding a response rate of 97.8%. The mean age of the participants was 20.12 years (SD ± 2.78). Among the participants, 379 (60%) were males. Five hundred and three (79.6%) of the participants were unmarried. The majority, 493 (78%), of respondents were orthodox in their religion. Three hundred eighty seven (61.2%) of participants were students. More than half (53%) of the participants' friends have had substance use history (Table 1).

### 3.2. Prevalence of Psychoactive Substance Use among Youths

Life time and current prevalence of any one of the psychoactive substances use were 66.1% and 65%, respectively. Almost half, 311 (49.2%), of the participants were current users of alcohol followed by khat current users 146 (23.1%). Fifty one (8.1%) and eighteen (2.7%) participants were current users of tobacco and cannabis, respectively. The life time prevalence of alcohol, khat, tobacco, and cannabis use were 50.3%, 23.3%, 8.2%, and 2.8%, respectively. There were different reasons reported by the participants to initiate using psychoactive substances. Among the participants; the most common reason was for relaxation 143 (44.96%), followed by family members use it 82 (25.8%), peer pressure 61 (19.2%), to relieve anxiety 20 (6.3%), and to kill time 12 (3.8%) (Table 2).

### 3.3. Factors Associated with Psychoactive Substance Use among Youths

Multivariate logistic regression analysis revealed that being male, with higher income, family members, and friends of the participants using substance were statistically significant with current use of psychoactive substance (*P* < 0.050). Males were twice more likely to be psychoactive substance users than females (AOR = 2.48; 95% CI: 1.45, 4.23). Higher income was responsible to cause psychoactive substance use, 6 and 20 times ((AOR = 5.92; 95% CI: 1.67, 20.95) and (AOR = 26.18; 95% CI: 2.70, 253.61)) with income of 1000–2000ETB and 2001–3000ETB more than for individuals with lower income (<1000ETB), respectively. History of substance use from respondents' family members and friends ((AOR = 2.00; 95% CI: 1.14, 3.51) and (AOR = 29.69; 95% CI: 15.82, 55.73)) were significantly associated with current psychoactive substance use, respectively, (Table 3).

## 4. Discussion

The life time and current prevalence of psychoactive substance use in this study were 66.1% and 65%, respectively. This result is higher than the research studies conducted in Ethiopia at different universities [29] (45.9% of life time use and 44.8% of current use), [30] (53.6% of life time and 35.5% current use), [31] (overall prevalence of 32.28%), [32] (48.4% of life time prevalence), and [33] (ever substance use of 45.5%). It was also higher than the research conducted in Rwanda [34] which was 52.5%, and in Nigeria among undergraduates in the University of Uyo which was 27.5% [35]. Current alcohol use (49.2%) was the most commonly used substance which was followed by khat (23%). It was also higher than a community based study conducted at Jimma town on street children age 12–18 years old which was 30.8% [36]. There were two previous studies in Zaira, Nigeria [37] and Kenya university [38], those had higher prevalence (69.3% and 69.8%, respectively) of psychoactive substance use than the current study (65%). The discrepancies occurred among the current and previous studies could be due to different assumptions. The most probable cause of the difference might be due to the study settings on which the studies were conducted. Our current study was conducted at community level which was not in many of the previous studies which all have focused in institutions. The other reason of this difference might be due to the study tools we used. In this study, we have applied the ASSIST tool which was rarely used in other previous studies we have seen. Age difference of the participants might be another possible reason for the difference. However, the prevalence of this study was almost in line with the previous study conducted in Raikarpada (64.9%), a state of India [39].

Current alcohol use (49.2%) in this study was the most common substance used by the study participants which was followed by current khat (23.1%) and tobacco use (8.1%). This result is higher than the results of previous studies conducted in Ethiopian University under graduate students [29, 40, 41]. In that previous study the current prevalence of alcohol drinking was 32.8% which was lower than the current result (49.2%). But, the previous study's result for khat chewing was 27.9% and cigarette smoking was 9.3% which were greater than the results of the recent study which were 23.1% and 8.1%, respectively. This discrepancy might be due to the difference in study settings that the previous study was institution based whereas the current was community based.

The result of previous study conducted at Adigrat university [42] showed that students on current chewing of khat and cannabis use were 34.2% and 32.3%, respectively, which were higher than the results of current study to mentioned substances; however, current alcohol drinking by the study participants in our research was 49.2%, which was higher than the study result (29.2%) found In Adigrat Univesity. The study settings and the geographical differences which contributed for the availability of substances might cause the difference.

An institution based research conducted at Haromaya university [43] in Ethiopia showed that the current prevalence of alcohol use was 20% which was much lower than the current study (49.2%). The prevalence of chewing of khat in mentioned university was 23.6%, almost the same as the current study (23.1%), but prevalence of current cigarette smoking in previous study was 10.8% which was slightly higher than the current study (8.1%). The differences found in these two studies might be due to the study settings, location, and the tool differences.

Another study conducted at Addis Ababa university [44] students showed that the current prevalence of alcohol, khat, and cigarette smoking were 9.3%, 3.7%, and 1.8%, respectively, which was extremely lower than the findings in current study. The study settings, duration of the study, and the tools used might cause this gap.

Being male was positively associated with current use of psychoactive substance. It was two times more likely to be used by male participants than the female ones. This is also true in most of the previous studies conducted in Ethiopian public university students [17, 29, 30, 32, 40, 41, 43, 44] and in other World countries [34, 35, 38, 45, 46]. This could be due to the fact that most of the Ethiopian cultures allowed (assumed) to reinforce males to use substances such as alcohol and khat. On the other hand, the norm discourages females especially young females not to take any substance including alcohol. The other reason might be due to the fact that boys are less likely to control their emotion than girls who always trying to experiment things which are new for them whether the event is harmful or useful without any hesitation.

History of family members and friends' substance use were positively associated with current substance use of the participants of our study. This might be due to the principle of “modeling” or “learning by seeing.” That means modeling is the main reason to begin and repeat a new behavior to experiment why and how others are using for. This type of learning from others within the circumstances of socialization is strongly applied in young people. This was also in line with different studies conducted in Ethiopia [29, 36, 41, 44] and in other African countries [34, 35, 46, 47].

The other important factor found positively associated with current substance use in our study was having of higher monthly income. This means, having highly income might allow people to exercise sedentary life style. This might be due to the fact that when someone became rich; there would be an opportunity to do what the individual needs including recreational activities such as psychoactive substance use.

## 5. Conclusion

The prevalence of life time and current psychoactive substance use was high as compared to other studies conducted in Ethiopia. This shows that most of the young people, who will be the future productive engine of the nation, are involved in dangerous substances responsible to change the structure and function of the brain which makes them pessimistic. Being male, high income, family members, and friends of participants using psychoactive substance increased the odds of current psychoactive substance use. Thus, concerned stakeholders should collaborate on awareness creation and policy revision about the law towards psychoactive substances.

## 6. Limitations of the Study

Since our study was cross-sectional, it is difficult to determine the temporal relationship between the outcome and the exposure, because both are examined at the same time. Without longitudinal data, it is not possible to establish a true cause and effect relationship.

## Figures and Tables

**Table 1:** Socio-demographic characteristics of study participants among youths at selected administrative towns, North Shewa Zone, Amhara regional state, Ethiopia, 2020 G.C.

Variables	Frequency (*n* = 632)	Percent (%)
*Sex*		
(1) Male	379	60
(2) Female	253	40

*Age in years*		
(1) 15–17	124	19.6
(2) 18–24	508	80.4

*Religion*		
(1) Orthodox	493	78
(2) Muslim	92	14.6
(3) Protestant	47	7.4

*Marital status*		
(1) Married	109	17.2
(2) Unmarried	503	79.6
(3) Divorce	20	3.2

*Educational status*		
(1) Not read and write	5	0.8
(2) Read and write only	64	10.1
(3) Primary school	118	18.7
(4) Secondary school	248	39.2
(5) College/university student	138	21.8
(6) Diploma and above	59	9.3

*Occupational status*		
(1) Governmental employed	42	6.6
(2) Merchant	43	6.8
(3) Student	387	61.2
(4) Housewife	21	3.3
(5) Daily laborer	80	12.7
(6) Unemployed	59	9.3

*Monthly income*		
(1) <1000ETB	482	76.3
(2) 1000–2000ETB	48	7.6
(3) 2001–3000ETB	48	7.6
(4) >3000ETB	54	8.5

*Living conditions*		
(1) With family	176	27.8
(2) With relatives	314	49.7
(3) With friend	59	9.3
(4) Alone	83	13.2

*Family members substance use*		
(1) Yes	313	49.5
(2) No	319	50.5

*Friends substance use*		
(1) Yes	335	53
(2) No	297	47

**Table 2:** The prevalence of current and life time psychoactive substance use among youths in selected administrative towns, North Shewa zone, Amhara regional states, Ethiopia, 2020.

Characteristics	Sex		Total
	Male	Female	
	Yes *n* (%)	Yes *n* (%)	Yes *n* (%)
	No *n* (%)	No *n* (%)	No *n* (%)
Ever psychoactive substance use	283 (44.8)	135 (21.3)	418 (66.1)
	96 (15.2)	118 (18.7)	214 (33.9)

Current psychoactive substance use	278 (44)	133 (21)	411 (65)
	101 (16)	120 (19)	221 (35)

Ever alcohol drinking	225 (35.6)	93 (14.7)	318 (50.3)
	154 (24.4)	160 (25.3)	314 (49.7)

Current alcohol drinking	221 (35.0)	90 (14.2)	311 (49.2)
	158 (25.0)	163 (25.8)	321 (50.8)

Ever use of khat	95 (15.0)	52 (8.2)	147 (23.3)
	284 (44.9)	201 (31.8)	485 (76.7)

Current use of khat	94 (14.9)	52 (8.2)	146 (23.1)
	285 (45.1)	201 (31.8)	486 (76.9)

Ever smoking of cigarette	44 (7.0)	8 (1.3)	52 (8.2)
	335 (53.0)	245 (38.8)	580 (91.8)

Current cigarette smoking	43 (6.8)	8 (1.3)	51 (8.1)
	336 (53.2)	245 (38.8)	581 (91.9)

Ever use of cannabis	10 (1.6)	8 (1.3)	18 (2.8)
	369 (58.4)	245 (38.8)	614 (97.2)

Current use of cannabis	9 (1.4)	8 (1.3)	17 (2.7)
	369 (58.5)	245 (38.8)	614 (97.3)

**Table 3:** Factors associated with current psychoactive substance use among youths of age 15–24 years at three selected administrative towns, North Shewa Zone, Amhara regional state, Ethiopia, 2020 (*n* = 632).

Variables	Current psychoactive substance use		*P* < 0.25	COR (95% CI)	*P* < 0.05	AOR (95% CI)
	Yes	No				

*Age in years*						
(1) 15–17	51	73				1
(2) 18–24	360	148	*P* ≤ 0.001	3.48 (2.32, 5.22)	0.852	0.94 (0.50,1.77)

*Sex*						
(1) Male	278	101	*P* ≤ 0.001	2.48 (1.78, 3.47)	0.001	2.48 (1.45, 4.23)^*∗*^
(2) Female	133	120				1

*Marital status*						
(1) Married	92	17				1
(2) Unmarried	301	202	*P* ≤ 0.001	0.28 (0.16, 0.48)	0.272	0.59 (0.24, 1.49)
(3) Divorced	18	2	0.520	1.66 (0.35, 7.83)	0.638	1.61 (0.22, 11.83)

*Income*						
(1) <1000ETB	277	205				1
(2) 1000–2000ETB	42	6	*P* ≤ 0.001	5.18 (2.16, 12.42)	0.006	5.92 (1.67, 20.95)^*∗*^
(3) 2001–3000ETB	47	1	*P* ≤ 0.001	34.78 (4.76, 254.18)	0.005	26.18 (2.70, 253.61)^*∗*^
(4) >3000ETB	45	9	0.001	3.70 (1.18, 7.74)	0.344	2.35 (0.40, 13.71)

*Family member substance use history*						
(1) Yes	261	52	*P* ≤ 0.001	5.66 (3.91, 8.19)	0.015	2.00 (1.14, 3.51)^*∗*^
(2) No	150	169				1

*Friend substance use history*						
(1) Yes	316	19	*P* ≤ 0.001	35.36 (20.96, 56.68)	*P* ≤ 0.001	29.69 (15.82, 53.73)^*∗*^
(2) No	95	202				

Note: ^*∗*^Statistically significant.

## Data Availability

All the data used to support the findings of this study are included within the article.

## Abbreviations

AOR: Adjusted odds ratio
ASSIST: Alcohol, smoking, and other substance involvement screening test
COR: Crude odds ratio
CNS: Central nervous system
DALYs: Disability adjusted life years
DBU: Debre Berhan university
ETB: Ethiopian birr
WHO: World Health Organization.
